# Ultrathin and multicolour optical cavities with embedded metasurfaces

**DOI:** 10.1038/s41467-018-05034-6

**Published:** 2018-07-10

**Authors:** Amr M. Shaltout, Jongbum Kim, Alexandra Boltasseva, Vladimir M. Shalaev, Alexander V. Kildishev

**Affiliations:** 1 School of Electrical & Computer Engineering and Birck Nanotechnology Center, Purdue University, West Lafayette, Indiana 47907 USA; 2 Geballe Laboratory for Advanced Materials, Stanford University, Stanford, CA 94305 USA; 3 Institute for Research in Electronics and Applied Physics, University of Maryland, College Park, Maryland 20742 USA

## Abstract

Over the past years, photonic metasurfaces have demonstrated their remarkable and diverse capabilities in advanced control over light propagation. Here, we demonstrate that these artificial films of deeply subwavelength thickness also offer new unparalleled capabilities in decreasing the overall dimensions of integrated optical systems. We propose an original approach of embedding a metasurface inside an optical cavity—one of the most fundamental optical elements—to drastically scale-down its thickness. By modifying the Fabry–Pérot interferometric principle, this methodology is shown to reduce the metasurface-based nanocavity thickness below the conventional *λ*/(2*n*) minimum. In addition, the nanocavities with embedded metasurfaces can support independently tunable resonances at multiple bands. As a proof-of-concept, using nanostructured metasurfaces within 100-nm nanocavities, we experimentally demonstrate high spatial resolution colour filtering and spectral imaging. The proposed approach can be extrapolated to compact integrated optical systems on-a-chip such as VCSEL’s, high-resolution spatial light modulators, imaging spectroscopy systems, and bio-sensors.

## Abstract (web summary)

Achieving miniature and versatile nanophotonic devices with metasurfaces is of great interest. The authors embed a metasurface inside an optical cavity to reduce thickness and provide degrees of freedom for controlling resonant wavelengths, enabling multi-band filtering, structural colouration and spectral imaging.

## Introduction

Photonic metasurfaces^1–6^ have enabled a new paradigm of controlling light by passing it through planar arrays of nanostructured antennas to induce a swift change in phase and/or polarization. Consequently, photons propagating through metasurfaces could be ‘processed’ to undergo a change to their momentum, angular momentum, and/or spin state. This has led to a relaxation of Snell’s law^2^, a pivotal relation in optics, and has enabled an entirely new family of flat optical elements. These elements can provide diverse functionalities for a plethora of applications including light bending devices^2,7^, flat lenses^8–11^, holograms^12–17^, wave-plates^18–22^, as well as devices with chiral^23–28^ and bianisotropic^29,30^ optical responses. A typical metasurface generates its functional output in the far field, which requires a separation distance between the metasurface and the location of the detected output. Therefore, even though a given metasurface could be ultrathin, an integrated optical system that requires cascading of the metasurface with another optical component may not fully utilize its capability to reduce the overall dimensions of the system.

Optical cavities are the major components of lasers and other numerous interferometric optical devices, and minimizing the cavity dimensions below the diffraction limit is the key for many applications, including realization of nano-lasers^31–33^, and the enhancement of spontaneous emission rate due to the Purcell effect^34^. This enhancement, which is useful for single-photon sources^35,36^ and low threshold lasing^37–39^ and is inversely proportional to the volume of the cavity, has motivated nanophotonics researchers in quest of solutions minimizing the cavity dimensions^40–44^.

Here we show that embedding a metasurface inside a most fundamental optical element—an optical cavity—can substantially reduce the cavity thickness (up to two times). Such significant reduction in the transverse dimension is achieved without changing the optical mode for the sake of cascading and integration with other optical devices. In the following sections, we present a metasurface-based Fabry–Pérot nanocavity arranged of two parallel reflective metal layers and a metasurface sandwiched between them. First, we theoretically study the impact of the metasurface on the resonant condition of nanocavity based upon the interferometric principle. Then, we discuss the structure fabrication and the experimental results, which show that a 100-nm nanocavity supports resonances within the wavelength band from 500 to 800 nm and demonstrate successful application of the fabricated nanocavity samples as on-a-chip ultrathin colour filters. In addition to reducing the cavity thickness, the metasurface can also add another degree of freedom to control the cavity resonance. With the introduction of the metasurface, the cavity resonance can be tuned through adjusting the parameters of the metasurface—not only by adjusting the cavity thickness. This is especially useful for building multiple cavities of the same thickness on a planar chip resonating at different wavelengths. These additional degrees of freedom also allow us to obtain dualband resonances or even produce coloured images by using a single planar nanocavity of the same thickness by simply choosing the appropriate design for the internal metasurface.

## Results

### Theoretical formulation

The resonant condition of a conventional Fabry–Pérot cavity made of two reflecting mirrors occurs when the round-trip optical phase inside the cavity is an integer multiple of 2π. This imposes a limitation on the cavity thickness to accumulate the required phase. Our approach to overcome this limitation is to place a metasurface inside the two mirrors which induces a strong phase shift and compensates for the reduced accumulated phase in the rest of the cavity.

**Fig. 1:**
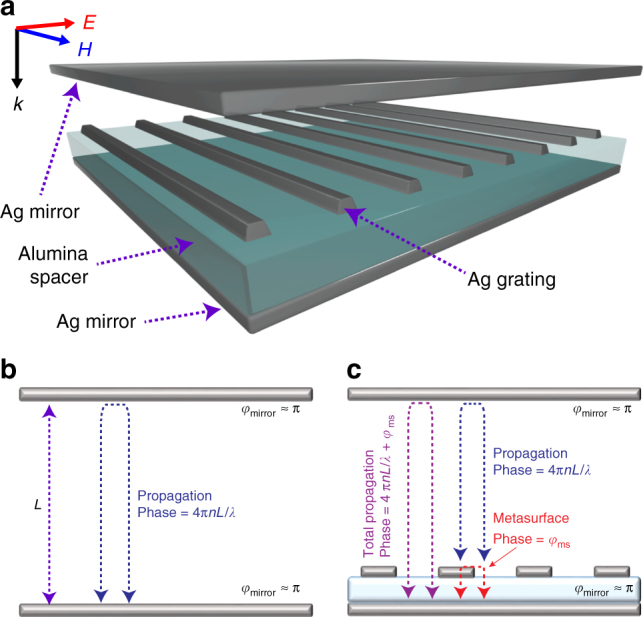
Design and concept of metasurface-based nanocavity. **a** Schematic of the metasurface-based nanocavity. **b**, **c** Comparison in phase and resonance conditions between: **b** conventional Fabry–Pérot resonator that utilizes two parallel mirrors and **c** resonator with a reflecting metasurface placed in between the two mirrors

Figure 1(a) is a schematic of the nanocavity composed of two silver layers as reflecting surfaces, a silver-based metasurface separated from the bottom Ag mirror by an alumina spacer layer, and a polymer which fills the rest of the cavity. Figure 1(b, c) demonstrate the cross section of a Fabry–Pérot cavity without and with the embedded metasurfaces, respectively. At resonance, the round-trip phase shift inside the cavity satisfies the following formulas in case of utilizing a perfect mirror:

1 $$\begin{array}{*{20}{l}} {\frac{{4{\uppi}nL}}{\lambda } = 2{\uppi}m,\;{\mathrm{without}}\;{\mathrm{metasurface}},} \hfill \\ {\frac{{4{\uppi}nL}}{\lambda } + \varphi _{{\mathrm{ms}}} = 2{\uppi}m,\;{\mathrm{with}}\;{\mathrm{metasurface}}} \hfill \end{array}$$

where *L* is the thickness of the cavity, *n* is the refractive index of the filling material, *φ*_ms_ is the phase shift induced by the metasurface, and $m = 1,2, \ldots$. The conventional formula implies that for the metasurface-free cavity, a minimum cavity thickness *L*_min_ is on the order of *λ*/(2*n*) when *m* = 1. In the case of an embedded metasurface, the induced phase *φ*_ms_ reduces the required phase 4π*L*/*λ* for the rest of the cavity, and hence enables *L*_min_ to go below *λ*/(2*n*). For practical purposes of operating with a very thin silver layer, a model, which includes an extra phase term induced by the Ag mirror, is more accurate^45^

2 $$\begin{array}{*{20}{l}} {\frac{{4{\uppi}nL}}{\lambda } + \varphi _{{\mathrm{Ag}},1} + \varphi _{{\mathrm{Ag}},2} = 2{\uppi}m,\;{\mathrm{without}}\;{\mathrm{metasurface}}} \hfill \\ {\frac{{4{\uppi}nL}}{\lambda } + \varphi _{{\mathrm{Ag}},1} + \varphi _{{\mathrm{ms}}} = 2{\uppi}m,\;{\mathrm{with}}\;{\mathrm{metasurface}}} \hfill \end{array}$$

where $\varphi _{{\mathrm{Ag}},1}$ and $\varphi _{{\mathrm{Ag}},2}$ are the extra phase shifts induced by the top and bottom silver mirrors, respectively, for $m = 1,2, \ldots$. In case of having the metasurface embedded with the bottom mirror, the term $\varphi _{{\mathrm{Ag}},2}$ should be replaced by $\varphi _{{\mathrm{ms}}}$. In the case of 15-nm silver Ag mirror, studies have shown^45^ that phase shift induced by silver mirrors would make the minimum cavity thickness *L*_min_ on the order of 0.35*λ*/*n*.

The underlying physical mechanism that induces the strong phase shift by the metasurface is the excitation of gap-plasmon resonators^46–48^ and slow plasmonic modes that propagate inside the alumina spacer layer between the bottom silver layer and the metasurface structure. These gap-plasmon modes can create large phase shifts for the wavefronts propagating inside the cavity. The phase shifts induced by the gap-plasmons can be controlled by adjusting the lateral dimensions of the metasurface Ag nano-strip layer. For example, increasing the nano-strip width makes the gap-plasmons propagate over a greater lateral distance and acquire additional phase, thereby decreasing the minimum cavity phase shift required for the Fabry–Pérot resonance^48^.

### Implementation and experimental results

**Fig. 2:**
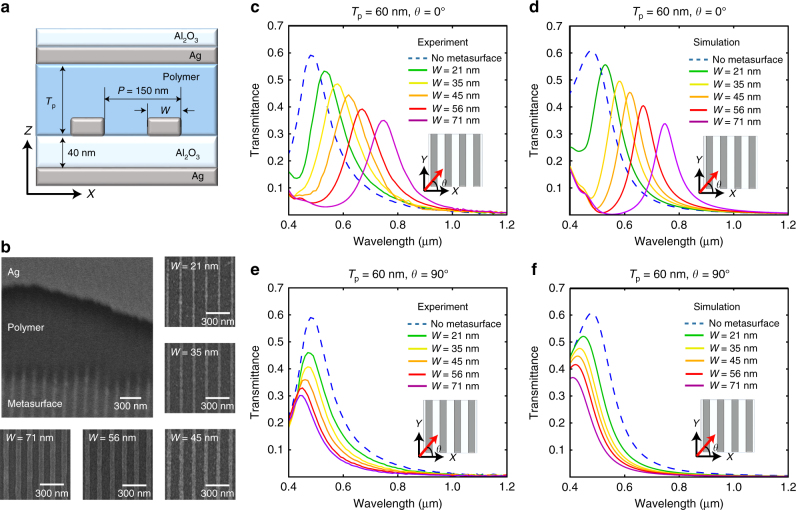
Spectral transmission of Ag based nanocavities with and without the metasurface. **a** Cross-sectional schematic of the metasurface-based nanocavity. *P* and *W* are the perodicity and linewidth of Ag grating, respectively. *T*_p_ is the thickness of polymer layer. The thickness of both top and bottom Ag mirror is 15 nm and the thickness of grating is 22 nm. **b** 30° tilted view and Top view of FE SEM (Field Emission Scanning Electron Microscopy) image of fabricated nanocavity. Experiment (**c**, **e**) and simulation (**d**, **f**) of transmission spectra of nanocavity with 60 nm thick polymer spacer (inset in **c**: the schematic of polarization direction, *θ* is the angle of polarization of incident wave): **c**, **d** *θ* = 0° and **e**, **f** *θ* *=* 90°

Figure 2(a) shows a cross-sectional schematic of the nanocavity. It is implemented with 15-nm-thick Ag mirrors, a 40-nm-thick Alumina spacer, 22-nm-thick Ag metasurface gratings, and a polymer thickness in the rest of the cavity of 60 nm, which makes the whole cavity thickness between the two mirrors to be around 100 nm. The structure has a lateral period of 150 nm. Figure 2(b) shows a 30° tilted view of the nanocavity where a cross-sectional shear is introduced through the upper Ag layer and the polymer layer till the metasurface layer. Five different samples are fabricated with different metasurface widths of *W* *=* 21, 35, 45, 56, and 71 nm. All the samples are fabricated on the same chip, and hence, they hold the same cavity thickness and any difference they exhibit in their optical performance is attributed to the different width (*W*) in their metasurface structure. (See Methods section for the detailed fabrication and measurement procedure).

To demonstrate the impact of the metasurface on the nanocavity, spectral transmission measurements are conducted on the nanocavities with and without the metasurface. Figure 2(c, d) demonstrate spectral measurements and the transmission data respectively, obtained from finite-element simulation with the incident polarization being perpendicular to the Ag metasurface gratings, since this polarization enables gap-plasmon excitation across the width of the meta-gratings^48^. Comparing the spectral data without a metasurface (dashed line) to those of metasurface-based nanocavities, we conclude that the metasurface makes the resonant wavelength longer across the visible and NIR spectra. A numerical study of the phase shift induced by the metasurface is included in Supplementary Fig. 1. The study provides that increasing the metasurface Ag grating dimensions increase the induced phase shift (*φ*_ms_) inside the metasurface. It is demonstrated in Supplementary Fig. 1 that *φ*_ms_ has a broadband response. This is owing to the fact that the retardation phase in the gap-plasmon structure is not a resonant effect. The relatively large 40-nm thickness of the spacer is responsible for coupling most of the optical power to the gap-plasmon mode. If the spacer thickness were too narrow, the light would only couple to the gap-plasmon mode when Fabry–Pérot resonance overlapped with gap-plasmon resonance creating a hybrid mode^49^, which would be different from the effect required in our case, moreover it would demand very careful adjustment of the structure to match the Fabry–Pérot and plasmonic resonances, otherwise, it would not operate.

According to Equation (2), increasing *φ*_ms_ causes an increase in wavelength *λ* for the equation to be satisfied. Therefore, resonant wavelength increases as the Ag gratings width increases across the values *W* *=* 21, 35, 45, 56, and 71 nm. All these samples resonating at different wavelengths are having the same cavity thickness. The structured metasurface adds a new degree of freedom of controlling resonant wavelength other than the cavity thickness. On the other hand, the impact of the metasurface on the resonant wavelength when light polarization is parallel to the gratings is not significant since this polarization is poorly coupled to the gap-plasmonic mode. This reflects in the spectral measurement and simulation data shown in Fig. 2(e, f). The simulation data is well-matched with the experimental data; however, the simulated transmission data within the wavelength range from 400 to 500 nm are higher, since the utrathin Ge adhesion layer is excluded from the simulation domain^50^. Supplementary Fig. 2 demonstrates the simulation of the reflection and the absorption spectra to complement the transmission study in Fig. 2(d). The dominant loss mechanism is attributed to the absorption in the metal mirror that can be shown by comparing the absorption of devices without and with the metasurface. The increase of absorption due to the insertion of the metasurface is insignificant (below 10%). Despite the relatively low additional loss due to the metasurface, it has a wideband effect due to the strong coupling efficiency in and out of the gap-plasmon mode. Another experimental study on a different metasurface-based nanocavity of polymer thickness 240 nm is demonstrated in Supplementary Fig. 3. It shows that the impact of the metasurface in increasing the resonant wavelength is effective in fundamental resonant mode as well as higher order modes.

### Dualband resonance

The formula of Equation (2) as well as the measurements in Fig. 2(c) indicates that metasurface-based nanocavities are dependent on both the cavity thickness *L* and the metasurface induce phase *φ*_ms_, making the resonance controllable via two independent degrees of freedom. If the metasurface structure is designed such that it can induce a strong phase shift *φ*_ms_ at more than one wavelength, then multiple resonances can occur at different wavelengths that can be independently controlled through careful design of the metasurface. This enables a new technique of obtaining multi-band resonance other than having different higher modes standing waves across the cavity thickness (modes with *m* > 1).

**Fig. 3:**
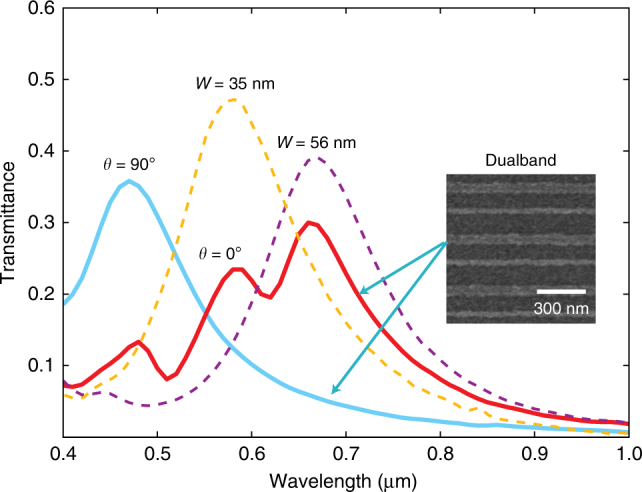
Spectra transmission of dualband nanocavity. Experimentally obtained transmission spectra of dualband nanocavity with 60-nm-thick polymer spacer: the dualband metasurface consists of two different Ag gratings with 35 nm and 56 nm linewidth

To obtain a dualband resonance, we implement a nanocavity where the metasurface is composed of alternating grating widths of 35 and 56 nm as shown in Fig. (3). We have had two samples, each of them has its grating width designed with only one of these two values. They were part of the samples whose results are shown in Fig. 2. We demonstrate the individual response of each of these two samples in Fig. 3 along with the sample implemented with the alternating grating. The corresponding simulation results are presented in Supplementary Fig. 4. The sample with the hybrid Ag grating dimensions have demonstrated dualband resonances, which coincide with the two individual resonances obtained by the other two samples. This can be attributed to the metasurface’s hybrid dimensions which will support gap-plasmonic modes at both wavelengths supported by grating widths of 35 and 56 nm. The small peak at a wavelength similar to the resonance of the cross-polarized light is attributed to the strong absorption of Ge layer within the wavelength from 400 nm to 500 nm (See Supplementary Fig. 4). The different resonant wavelengths obtained through this technique can be independently tuned by a separate adjustment of different grating widths.

### Ultrathin colour filtering and imaging

**Fig. 4:**
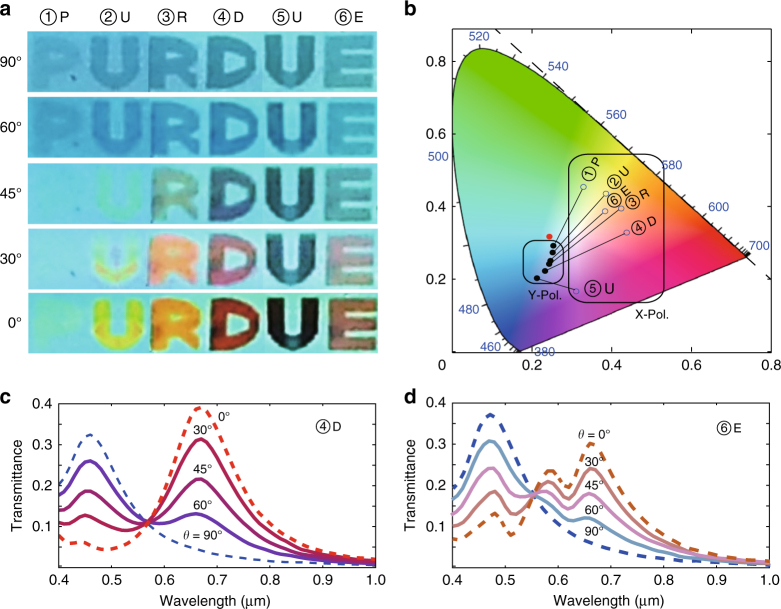
Bandpass colour filtering with the metasurface-based nanocavities in the visible range. **a** Colour image of the nanocavity with different linewidth. White light illuminates the sample via a linear polarizer. The angle of polarization is varied from 0° to 90°. **b** The variation of colour with angle of polarization is analyzed with the CIE 1931 colour mapping. The red dot represents the background colour. **c**, **d** The transmission spectra of nanocavity at different angle of polarization: **c** for sample 4 (letter ‘D’) and **d** for sample 6 (letter ‘E’)

One of the straightforward and promising applications of optical cavities is spectral filtering. The polarization sensitive colour filtering can be obtained from the metasurface embedded nanocavity due to its narrow linewidth and broad tunability of resonance in the visible range. In addition, from white light through corresponding shaping of nanocavities, colour images can be generated in planar surface which make this device easy to be integrated with other optical components. With the same nanocavity thickness of 100 nm as in previously shown results, we implement six different nanocavities, each of them is shaped to one of the letters of the word PURDUE, with each letter having a dimension of 500 µm × 420 µm. The letters P, U, R, D, U are shaped with Ag metasurfaces having grating widths of *W* *=* 21, 35, 45, 56, and 71 nm, respectively, which are the same dimensions of metasurfaces as demonstrated in Fig. 2. Letter E is implemented using a metasurface with alternating gratings of 35 and 56 nm similar to the one in Fig. 3. Broadband light from a white lamp is filtered through a polarizer, and applied to the sample, and multi-coloured images are captured from the other side of the sample. Figure 4(a) demonstrates the colour images obtained for different polarization directions of incident light with 0° corresponding to perpendicular polarization, and 90° to parallel polarization with respect to Ag gratings. The coloured image is in good agreement with the spectral data in Figs 2–3. First, the part of the cavity outside the metasurface filters a sky-blue colour that corresponds to its spectral data. The images of samples 1, 2, 3, and 4 for 0° polarization have their resonant wavelengths pushed to the spectra of green, yellow, orange, and red, respectively. For sample 5, the spectral data is in the NIR outside the visible spectrum explaining the dark appearance of the letter E. For sample 6, there is a dualband (such as the one in Fig. 3) which is a hybridization of two colours leading to the generation of the brown colour for letter F. For all the six samples, the 90°-polarized light generates a blue colour similar to the one in the metasurface-free background with a smaller intensity. This is also in agreement with the experimental data shown in Fig. 2. For other polarizations between 0° and 90°, the obtained spectrum is a linear combination of the horizontally and vertically polarized spectra. Figure 4(c, d) present the measured spectra for these polarizations for sample 4 and 6. In order to understand what colour will be the result of this linear superposition, we need to use the standard 1913 CIE (Commission internationale de l'éclairage) colour map that maps the spectral data onto a corresponding colour. This map exhibits the property that a linear superposition of two spectra will correspond to the linear superposition of their corresponding locations on the map (i.e., along the line connecting the two locations). The map location of the six samples for horizontal and vertical polarizations were obtained (The methods section explains the tools used to obtain them). The line connecting the two locations in the map should reflect the colours generated when the polarization is rotated between 0° and 90°. Figure 4(a, b) indicate a good agreement between the colour images and the corresponding position on the 1931 CIE colour map. Previous implementations of colour filtering using metasurfaces^51–53^ depend mainly on plasmonic spectral absorption providing a band-stop filtering operation. This can be useful with colour displays working with C-M-Y-K basis, while our metasurface-based Fabry–Pérot structure provides bandpass colour filtering, which is more compatible with the most common R-G-B colour displays.

In addition to compactness, metasurface-based nanocavities offer more degrees of freedom to the design of colour filters and displays with respect to conventional metal-insulator-metal (MIM) cavities. For example, they can provide dualband operation which is beneficial for mixing up colours. Another key advantage is the possibility of building different cavities with different resonant wavelengths/colours within the same planar structure in one fabrication run. To demonstrate this capability, we realize the word 'PURDUE' with letters in colour on a single substrate by using a single EBL step allowing to build all letters simultaneously by varying the lateral dimensions of the metasurfaces. If similar cavities were to be realized using separate MIM cavities it would require six different cavity thicknesses or different filling materials, entailing a fabrication process, which would be substantially more challenging and less robust. In addition, the proposed design can be easily integrated with modern highly efficient broadband light emitters, providing a straightforward approach to building a multicolour display pixel on a single emitter.

## Discussion

A unique methodology of utilizing metasurfaces to achieve miniaturized nanophotonic devices is introduced. Metasurface-based nanocavities are proposed with dimensions that can go below the conventional diffraction limit of *λ*/(2*n*) in the material between the cavity mirrors. Embedding metasurfaces inside cavities provides other degrees of freedom for controlling resonant wavelengths in addition to adjusting the cavity thickness or dielectric material index. Several cavities are implemented on the same planar chip with the same thickness and we are able to obtain different resonant wavelengths through controlling the width of nanostructured metasurface elements. Multi-band filtering, colouration, and imaging is also made possible inside the same cavity by controlling the degrees of freedom provided by the metasurface. The proposed approach could have key impact for many optical applications including, but not limited to, nano-lasers and ultra-compact filters, as well as other interesting physical applications related to spontaneous emission enhancement based on the Purcell effect, cavity quantum electrodynamics, structural colouration, as well as plasmonics enhanced optoelectronic devices, including spatial light modulators with subwavelength resolution, spectroscopic imaging, and bio-sensing systems. Nano-lasers can be built by using a gain medium in the spacer and/or polymer layer. In addition, this work has the potential in future designs of dynamically controlled nanocavities utilizing tunable spacer layer materials, such as transparent conducting oxides, in which the free carrier concentration can be electrically or optically modulated. On the other hand, using a highly nonlinear spacer could be beneficial for optical limiting devices and saturable absorbers^54^.

## Methods

### Sample preparation

The designed structure is compatible with nanofabrication techniques. 15-nm-thick Ag and 40-nm-thick Al_2_O_3_ films were subsequently deposited on the glass substrate by electron beam evaporator. To fabricate Ag metasurface (grating structure), positive electron beam resist (ZEP 520 A) was spin-coated and grating arrays were defined by electron beam lithography (Vistec VB6) followed by lift-off process. On top of the patterned resist, 22-nm Ag film was deposited with a 3-nm Ge layer used to improve adhesion, and the sample was dipped in ZDMAC (dimethylacetamide) for 20 min. For planar surface of spacer layer on top of metasurface, we spin-coated 800 nm PMMA on top of metasurface and etched it down to desired thickness (60 nm or 240 nm) by reactive ion etching process (Panasonic E620 etcher). The roughness of etched PMMA spacer on top of metasurface is around 3 nm. Finally, 3-nm Ge film and 15-nm Ag film for top mirror layer and 40-nm alumina layer for protection of Ag were deposited on top of PMMA spacer.

### Sample characterization

The transmission spectra of fabricated nanocavities were measured using the spectroscopic ellipsometer (J. A. Woollam Co., V-VASE). The light source is a xenon lamp with a broadband Visible to NIR spectrum. The diameter of the incident beam was set to be 400 μm. The beam sequentially passed through a monochromator, a polarizer, and it is used to expose the sample, and then it’s collected by a detector. The colour image was captured with simple optical set-up. The white lamp was used as light source and linear polarizer was placed between lamp and sample. The image was captured with commercial camera in mobile phone (Samsung Galaxy S3) with ×4 magnification. ISO was set to 100, auto-contrast was off, and the micro lens was attached to camera.

### Simulation

We validated the experimental spectra with numerical simulations using a commercially available software (COMSOL Multiphysics, Wave Optics Module) based on the Finite Element Method. 1913 CIE colour map was calculated using Matlab with an open-access code from the Matlab website, https://www.mathworks.com/matlabcentral/fileexchange/29620-cie-coordinate-calculator.

### Data availability

The authors declare that the data supporting the findings of this study are available within the paper and its supplementary information file.

## Electronic supplementary material

Supplementary Information
