# Supplementary material for: Ultrathin and multicolour optical cavities with embedded metasurfaces
Source: Nat Commun. 2018 Jul 10;9:2673. doi: 10.1038/s41467-018-05034-6 (PMC6039493; doi:10.1038/s41467-018-05034-6)
Supplement: Supplementary file 1 — Supplementary Information [file 41467_2018_5034_MOESM1_ESM.pdf]

Ultrathin and Multicolour Optical Cavities with Embedded Metasurfaces

Amr M. Shaltout [1, 2, †], Jongbum Kim [1, 3, †], Alexandra Boltasseva [1],

*Vladimir M. Shalaev [1], and Alexander V. Kildishev [1, *]*

School of Electrical & Computer Engineering and Birck Nanotechnology Center, Purdue University, West Lafayette, IN 47907, USA

Geballe Laboratory for Advanced Materials, Stanford University, Stanford, California 94305, USA

Institute for Research in Electronics and Applied Physics, University of Maryland, College Park, Maryland 20742, USA

This document provides supplementary information to “Ultrathin and Multicolour Optical Cavities with Embedded Metasurfaces”.

Supplementary Note 1:

Supplementary Figure 1 shows the simulation data of phase-shift reflected from the metasurface based mirror with different metasurface dimensions compared to phase shift of perfect mirror. In this numerical study, the structure in fig 2(a) is simulated, with the upper silver mirror removed, and complex reflection coefficient from silver layer with and without the metasurface is calculated providing the value of the phase-shift. Another calculation is obtained by replacing the bottom silver layer with a perfect electric conductor (PEC) to provide a baseline reference of the phase-shift. The demonstrated phase-shift can determine the impact of the width of the metasurface elements (strips) on the resonance wavelength. With increasing the width, the phase-shift by

metasurface (φ_{ms}) is increased. Therefore, the resonance wavelength of nano-cavity is also increased with increasing the width of metasurface strips. In all simulations, Johnson-Christy model is used to model silver after multiplying the imaginary part of permittivity by 3 to account for extra loss of ultrathin and nano-structured silver compared to bulk silver. An electric permittivity of 2.9 is used to model alumina, and 2.1 to model polymer that is used to fill the cavity.

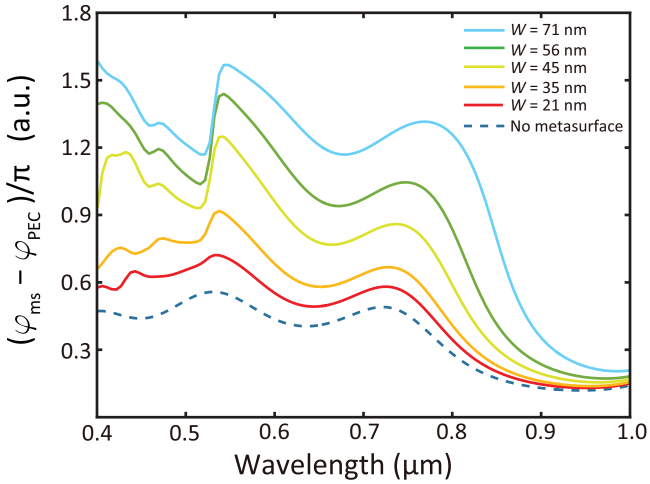

Supplementary Figure 1 Simulated phase of light reflected from the bottom silver mirror compared to the phase of light reflected from a perfect electric conductor ($\varphi_{ms} - \varphi_{PEC}$). The numerical value on the vertical axis represents the phase shift in terms of π (i.e., ($\varphi_{ms} - \varphi_{PEC}$)/π).

Supplementary Note 2:

Supplementary Figure 2 shows the simulation data of reflection and absorption spectra of the nanocavity with 60 nm PMMA and 40 nm Al_2O_3 layer as a spacer. The absorption of nanocavity mainly results from the optical loss in two Ag mirrors as shown in the absorption spectrum of nanocavity without metasurface. The metasurface also increases the absorption of nanocavity, but not significantly compared to the absorption from two mirrors.

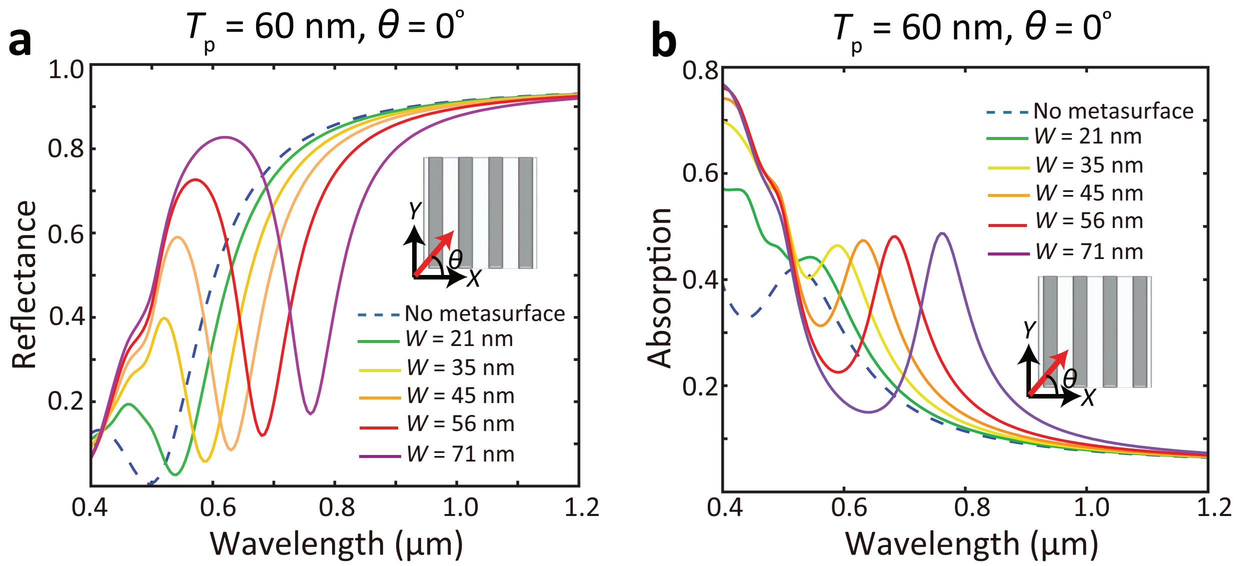

Supplementary Figure 2 Simulation of **(a)** reflection and **(b)** absorption spectra of nano-cavity with 60 nm thick polymer spacer (inset in (a) and (b): the schematic of polarization direction, θ is the incident light polarization angle).

Supplementary Note 3:

Supplementary Figure 3 shows transmission spectrum of the nanocavity with two orthogonal polarizations of incident wave. When the polymer thickness is 240 nm, the whole cavity thickness between the two metal mirrors becomes around 280 nm. In comparison with the nanocavity with 60-nm polymer layer, the 1st order cavity resonance is red-shifted to the near infrared regime and high order ($m > 1$) resonance appears in the visible range to satisfy the resonance conditions described in equation (2). Even though high order harmonic resonance is introduced by increasing the thickness of the polymer spacer, Ag metasurface causes the shift of all resonances because the induced phase shift due to metasurface (φ_{ms}) has the same impact on all orders of cavity resonances. Therefore, resonant wavelength increases as the width of the Ag metasurface strips increases across the values W = 21, 35, 45, 56, and 71 nm.

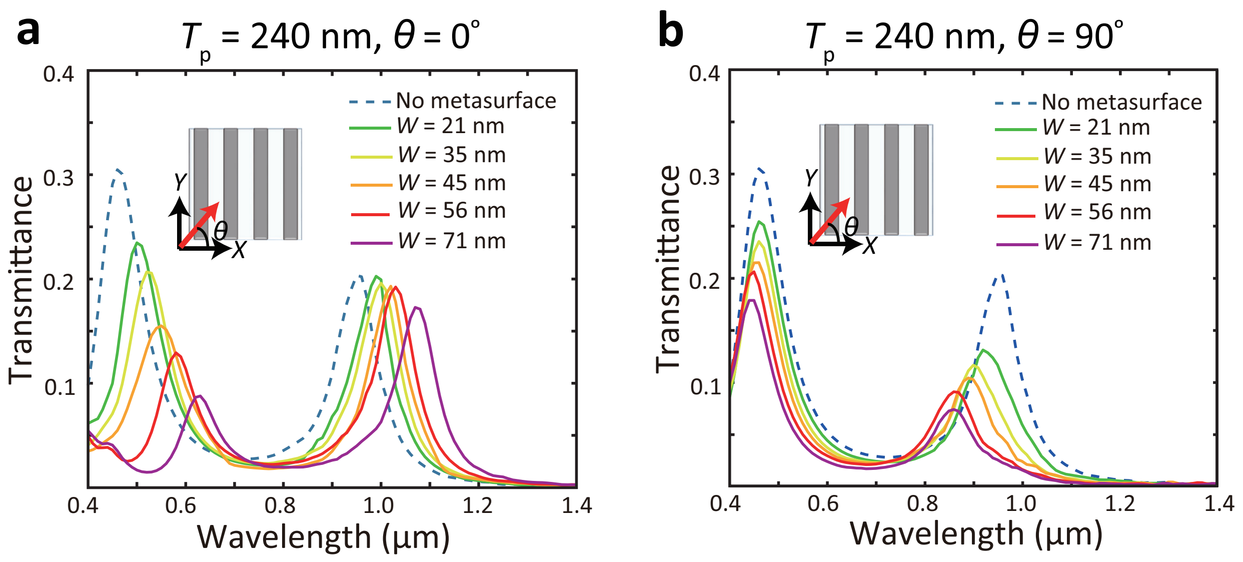

Supplementary Figure 3 Experiment of transmission spectra of nanocavity with 240-nm thick polymer spacer (inset shows the schematic of polarization direction, θ is the incident light polarization angle): **(a)** $\theta = 0°$ and **(b)** $\theta = 90°$.

Supplementary Note 4:

The simulation data of the nano-cavity where the metasurface is composed of alternating Ag strip gratings with the strip widths of 35 and 56 nm is shown in Supplementary Figure 4. As demonstrated in Fig (3), two resonances obtained by two different strip widths agree well with the individual response from each of these two Ag gratings with either 35 or 56 nm width. However, in comparison with the experiment data shown in Fig (3), the small peak at a wavelength of 480nm is not observed in the simulation data. Since the simulation does not include the Ge wetting layer that strongly absorbs the incident light within the wavelength range from 400 to 500 nm, we attribute the small peak at a wavelength of 480 nm observed in the experiment to the absorption of the Ge layer.

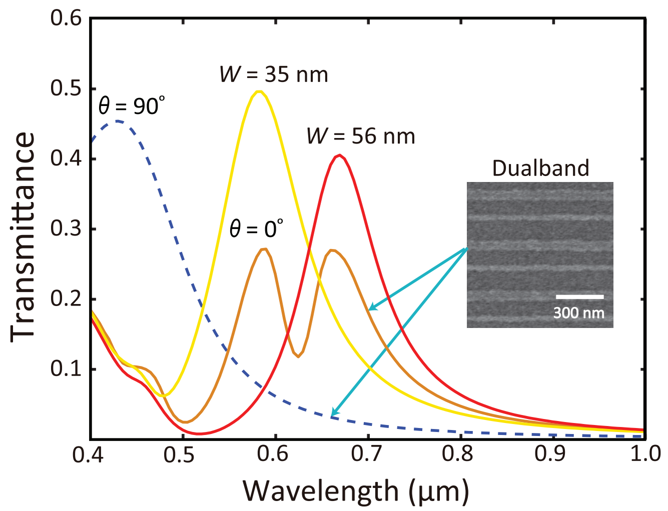

Supplementary Figure 4 Transmission spectra obtained from numerical simulations of the dualband nano-cavity with 60 nm thick polymer spacer: The dualband metasurface is arranged by combining two different periodic Ag strip gratings with the strip widths of 35 and 56 nm.
